# What radiologists need to know about 3D printing and its main applications in musculoskeletal imaging

**DOI:** 10.1590/0100-3984.2015.0208

**Published:** 2017

**Authors:** Francisco Abaeté Chagas-Neto, Francisco Coracy Carneiro Monteiro, Eduardo Lima da Rocha, Everaldo Gregio-Junior, Marcello Henrique Nogueira-Barbosa

**Affiliations:** 1 Centro Universitário Christus (Unichristus) e Hospital Antônio Prudente, Fortaleza, CE, Brazil; 2 Hospital Albert Sabin, Fortaleza, CE, Brazil; 3 Hospital Antônio Prudente, Fortaleza, CE, Brazil; 4 Faculdade de Medicina de Ribeirão Preto da Universidade de São Paulo (FMRP-USP), Ribeirão Preto, SP, Brazil

Dear Editor,

The utility of the various imaging methods in the evaluation and diagnosis of musculoskeletal disorders is well established, and those methods play a fundamental role in the planning of the different treatments, be they conservative or surgical, providing images that can be manipulated through specific software to create three-dimensional (3D) reconstructions. To date, however, such 3D reconstructions have been made available only as digital files, as images on radiographic films, or as prints on paper. These traditional forms of image documentation do not always allow surgeons to have a real in-depth sensory notion and knowledge of the 3D anatomical relationships in the planning of different types of surgical procedures. Recently, 3D printing has come to be used with increasingly frequency to obtain a more realistic and more accurate analysis by creating 3D models^(1-3)^.

What really is 3D printing? The definition of 3D printing, also known as rapid prototyping, is the use of a set of methods to create solid three-dimensional objects (models or prototypes) from the data contained in digital files. There are different forms of 3D printing, one of the most popular being the additive processing technique, in which the object is created layer by layer through successive depositions of a highly resistant plastic polymer.

How does 3D printing work? It all begins with the development of the 3D digital file. The file is obtained through the acquisition of sectional images through the use of magnetic resonance imaging, computed tomography, or even (3D or 4D) ultrasound. The digital file is then analyzed and processed with computer-aided design (CAD) software, according to what is required in each situation. After developing the 3D digital file, the CAD modeling software divides the prototype into hundreds or thousands of thin horizontal layers, thus preparing the file for printing. The digital file can then be loaded into a 3D printer for printing.

**Figure 1:**
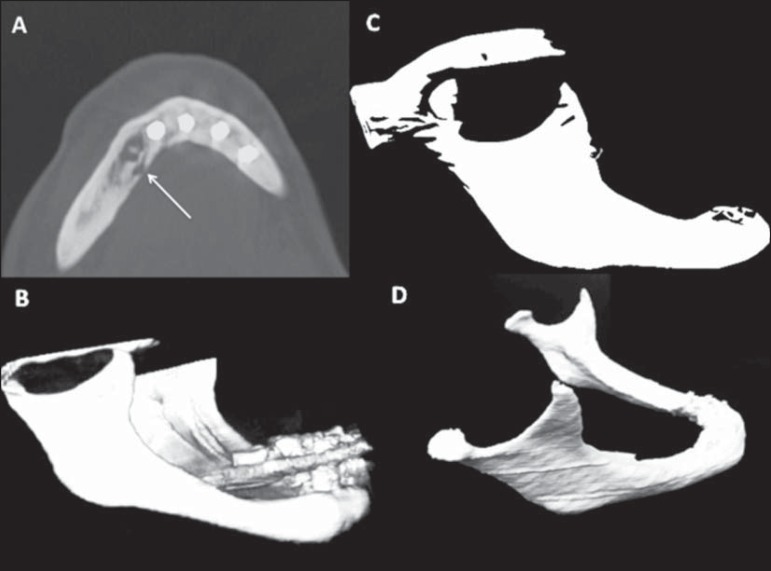
**A:** Axial computed tomography slice showing an osteolytic lesion in the right mandible (arrow). **B**: 3D volume-rendering computed tomography reconstruction of the mandible. **C**: 3D digital file, containing images of the mandible, being analyzed and processed in CAD software. **D**: Final aspect of the mandible prototype printed in 3D to mold the osteosynthesis material before surgery.

Is 3D printing already a reality in clinical practice or only in experimental research? In several countries, it is already a part of the clinical routine, having been shown to have a great impact on the precision and safety of surgical procedures^(2-5)^. There has been rapid growth in the number of potential applications of 3D printing in medicine, which has already been used in several situations, even in Brazil^(6-8)^. We illustrate, as an example, a case in which 3D printing was employed at our facility for the preoperative planning of the surgical treatment of an osteolytic lesion in the mandible (Figure 1).

What are the applications of 3D printing in musculoskeletal imaging? Among its many potential applications in clinical practice and in the teaching of medicine, we emphasize in this article the use of the technique in musculoskeletal imaging. We highlight its application in the preoperative planning of complex surgical procedures, which require high precision, such as those employed in the treatment of spinal deformities and complex fractures, as well as in the creation of models of orthotics and prostheses tailored to the anatomy and needs of each patient^(1-9)^.

We believe it to be inevitable that, in the coming years, there will be growth in the application of the 3D printing technique in the field of medicine as a whole, especially in the area of musculoskeletal imaging. The incorporation of this new technique will allow the optimization of protocols promoting good practices, offering greater effectiveness to the professionals involved and allowing better results, with potentially greater safety for patients.
